# IgG4-Related Disease Manifested as Hypertrophic Pachymeningitis: A Case Report and Literature Review

**DOI:** 10.3390/diagnostics16050682

**Published:** 2026-02-26

**Authors:** Xiao-Meng Liu, Li-Jun Yang, Lu Jin, Xiao-Lei Song, Jian-Liang Wu

**Affiliations:** 1 Department of Neurosurgery, The Second Hospital of Hebei Medical University, Shijiazhuang 050000, China; liuxiaomeng828@163.com (X.-M.L.); yanglijun1981@163.com (L.-J.Y.); songxiaolei92@163.com (X.-L.S.); 2 Department of Rheumatology and Immunology, The Second Hospital of Hebei Medical University, Shijiazhuang 050000, China; renxingyatou@126.com

**Keywords:** IgG4-related hypertrophic pachymeningitis, meningioma, serum IgG4, glucocorticoids, disease recurrence

## Abstract

**Background:** IgG4-related hypertrophic pachymeningitis (IgG4-RHP) is an extremely rare central nervous system (CNS) autoimmune disorder, characterized by dural thickening, space-occupying effects, and neurological compression symptoms. It is frequently misdiagnosed as meningioma due to overlapping radiological features, leading to inappropriate management. This study aims to report a unique case of IgG4-RHP with skull destruction and subcutaneous mass formation, and summarize its diagnostic and therapeutic strategies through literature review. **Methods:** A 53-year-old male with a chronic subdural hematoma history was admitted for a progressive right frontal subcutaneous mass. Preoperative computed tomography (CT) and magnetic resonance imaging (MRI) were performed, followed by staged surgeries (subcutaneous biopsy and craniotomy with subtotal resection). Histopathological examinations (Hematoxylin and Eosin staining, IgG/IgG4 immunostaining) and serum IgG4 detection were conducted. The patient received postoperative prednisone acetate (60 mg/d) and 3-month follow-up. A literature search was also performed to analyze 34 previously reported IgG4-RHP cases. **Results:** Histopathology showed dense lymphoplasmacytic infiltration, storiform fibrosis, ≈40 IgG4+ plasma cells per high-power field (HPF), and an IgG4+/IgG+ ratio of ≈30%. Serum IgG4 was significantly elevated to 1521 μg/mL (normal < 1350 μg/mL), with marked reduction in residual lesions on follow-up MRI. Literature review revealed a 73.5% male predominance, mean age of 48.6 years, headache as the most common symptom (58.8%), and a 38.5% misdiagnosis rate. Glucocorticoids alone or combined with immunosuppressants achieved favorable outcomes in 96.0% of treated cases. **Conclusions:** Histopathological examination combined with serum IgG4 detection is the gold standard for IgG4-RHP diagnosis. Surgical resection relieves mass-occupying effects, while glucocorticoids are first-line therapy. Long-term follow-up is necessary for recurrence monitoring, and rituximab is effective for refractory cases. Awareness of atypical manifestations like skull destruction can reduce misdiagnosis and improve outcomes.

## 1. Introduction

IgG4-related hypertrophic pachymeningitis (IgG4-RHP) is an exceptionally rare autoimmune disorder of the central nervous system (CNS), characterized by space-occupying effects and neurologic compression symptoms stemming from focal or diffuse thickening of the dura mater and spinal meninges [1]. Striking IgG4+ plasma cell infiltrate (IgG4+/IgG+ ratio > 40% or >10 IgG4+ plasma cells per high-power field [HPF]) and storiform fibrosis are major pathological hallmarks of IgG4-RHP. Serum IgG4 can be elevated in some IgG4-RHP patients [2,3]. IgG4-RHP can be easily misdiagnosed as meningioma due to the similar radiological features [3]. An early recognition of the autoimmune nature of IgG4-RHP, which is distinctly different from tumors, can avoid excessive surgery and guide hormone therapy. IgG4-related disease (IgG4-RD) is often seen in the pancreas, lungs and salivary glands. A single manifestation in the dura mater can be extremely rare. Isolated IgG4-RHP is a rare subtype of IgG4-RD. We reported a case of IgG4-RHP confirmed by pathological examination, presenting with the triad of “a history of chronic subdural hematoma + skull invasion + subcutaneous soft tissue mass”, which has not been documented in previous literature. The case was initially misdiagnosed as atypical meningioma and later confirmed by pathological re-examination of the resected mass. Postoperatively, the patient was treated with prednisone acetate at a daily dose of 60 mg, and significant regression of residual lesions was observed after 3 months. This study aims to provide clinical experience for the identification, diagnosis, treatment and management of this rare disease, reduce the rate of misdiagnosis, and improve patient prognosis.

## 2. Case Presentation

### 2.1. Clinical Presentation

A 53-year-old male presented with a 1-year history of a progressively enlarged subcutaneous mass in the right frontal lobe. Two years earlier, he underwent burr hole evacuation for a chronic subdural hematoma in the right frontotemporal lobe. Neurological and systemic examinations were unremarkable. A family history of autoimmune disorders or similar conditions in family members was not documented.

### 2.2. Imaging

**Figure 1:**
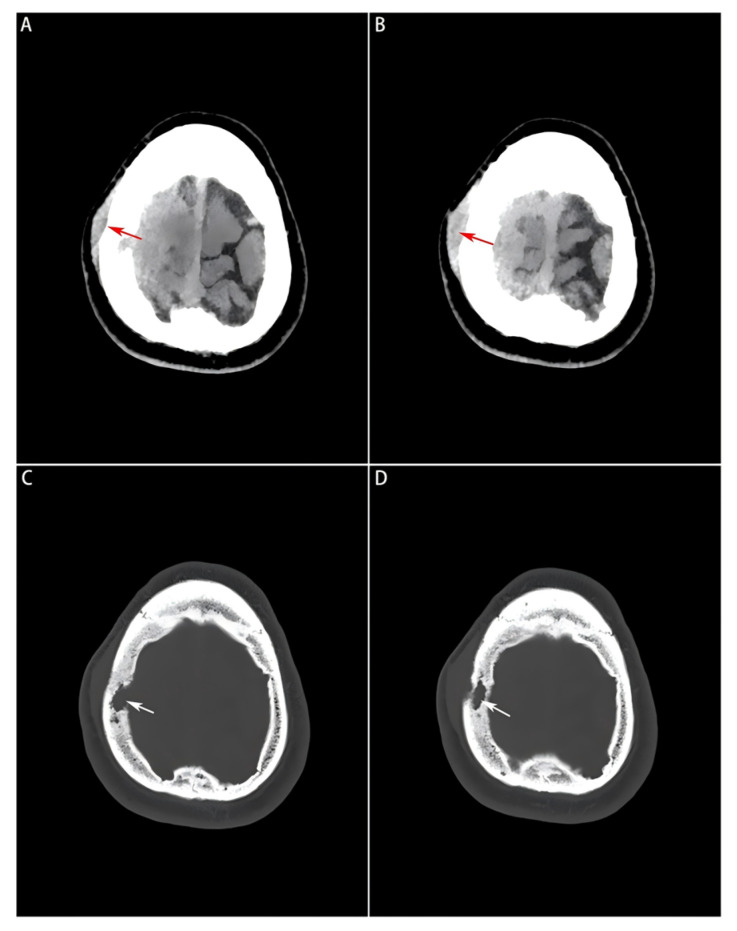
Preoperative head CT images of the soft tissue window and bone window. (**A**,**B**): A slightly high-density mass visualized on the soft tissue window, where the subcutaneous mass formed by the tumor lesion invading outside the bone window is indicated by a red arrow; (**C**,**D**): Bone erosion and defect visualized on the bone window image, and localized by a white arrow.

**Figure 2:**
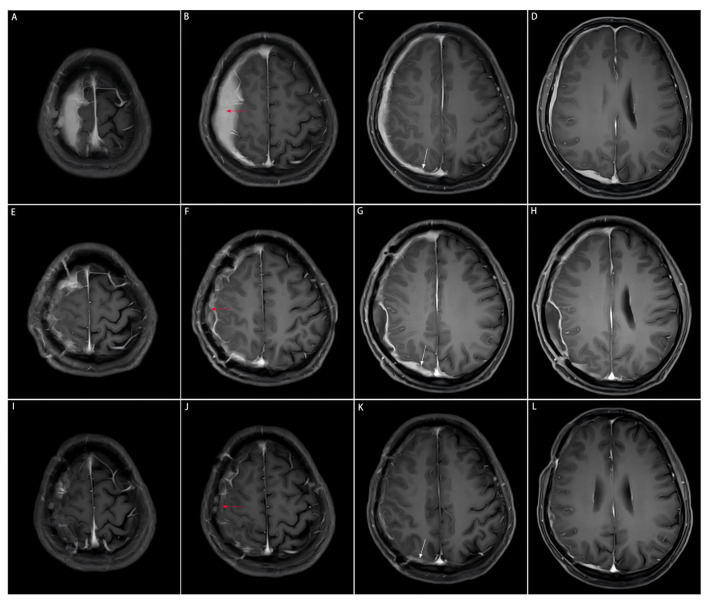
Comparison of preoperative, postoperative, and post-medication enhanced-contrast MRI images. The area of the resected dural mass before and after the subtotal resection, and after medication on enhanced-contrast MRI images is indicated by the red arrow. The dural mass close to the cerebral falx on enhanced-contrast MRI images is indicated by the white arrow. This part is not surgically removed due to the tricky localization approach to the midline, but significantly shrinks after the three-month medication of prednisone acetate. (**A**–**D**): Enhanced-contrast MRI images preoperatively, (**E**–**H**): 2 months postoperatively, (**I**–**L**): 3 months of prednisone acetate.

Head computed tomography (CT) scans (Philips Healthcare, Best, the Netherlands; software version R5.3) displayed a soft-tissue mass with a Hounsfield unit (HU) of 44, manifesting as a slightly high-density shadow in the right frontal lobe underneath the parietal bone, and compressed adjacent brain tissues (Figure 1A,B). Destruction of the right parietal bone was also seen on CT scans (Figure 1C,D). Nodular and strip-shaped thickening, uniform dural enhancement at the top of the right frontal, temporal and parietal lobes, and a spindle-shaped mass under the scalp with an abnormal cranial plate signal were found on preoperative contrast-enhanced magnetic resonance imaging (MRI) scans (Philips Healthcare, Best, the Netherlands; software version R6.1) (Figure 2A–D).

### 2.3. Surgical Interventions

**Figure 3:**
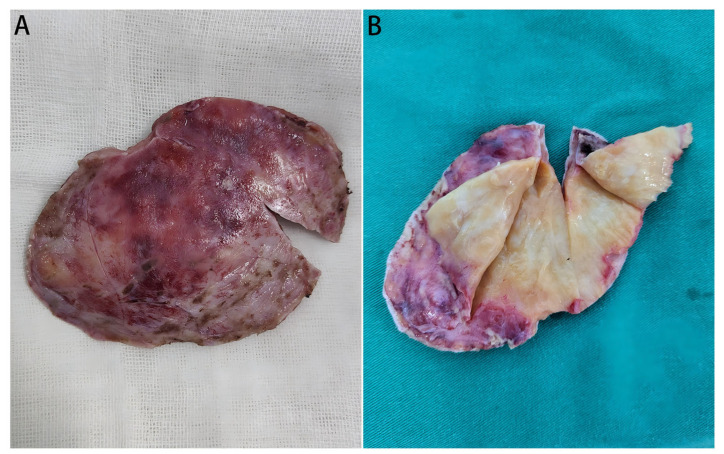
Gross views of the resected mass. (**A**): The overall view of the resected tumor mass; (**B**): Gross view the mass (cut open), which is yellow-white and tough, with fine textured internal tissues.

Staged surgeries were applied to the patient. First, the subcutaneous mass was biopsied for pathological examination, initially indicating a diagnosis of an atypical meningioma. A craniotomy was later performed, revealing a lesion invading two sites of the skull. The yellow-white, tough mass was located between the two layers of the dura mater without an invasion into the cerebral cortex, which was finally processed by a subtotal resection (Figure 3A,B). Contrast-enhanced MRI was performed during the follow-up period (Figure 2E–G).

### 2.4. Pathological Diagnosis

**Figure 4:**
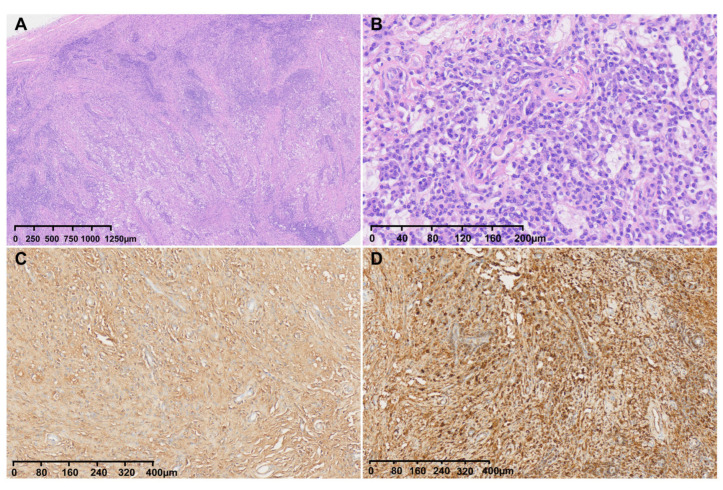
H&E and IgG/IgG4 staining of tumor sections. (**A**): H&E staining of tumors under low magnification; (**B**): H&E staining of the mass showing an abundant infiltration of plasma cells; (**C**): IgG staining of the mass; (**D**): IgG4 staining of the mass (about 40 IgG4+ plasma cells/HPF in the hot spots, and 30% of the IgG4+/IgG+ ratio).

In pathological examination, Hematoxylin and Eosin (H&E) staining of the intracranial specimen showed fibrosis, inflammatory infiltration of lymphocytes and plasma cells (Figure 4A,B), and elevated number of IgG4+ plasma cells (≈40 IgG4+ plasma cells/HPF, IgG4+/IgG+ ratio ≈ 30%) (Figure 4C,D). Finally, a pathological diagnosis of IgG4-RHP was confirmed. H&E Staining and IgG/IgG4 Immunohistochemistry: Specimens were fixed in 4% formaldehyde, embedded in paraffin, and serially sectioned at a thickness of 4 μm. H&E staining was performed using a standard hematoxylin-eosin staining kit (Manufacturer: Thermo Fisher, Waltham, MA, USA; Catalog No.: NC166). For IgG immunohistochemistry, a rabbit anti-human IgG monoclonal antibody was used (Clone No.: EPR4421; Manufacturer: Abcam, Cambridge, UK; Catalog No.: ab109489; Dilution ratio: 1:500). For IgG4 immunohistochemistry, a rabbit anti-human IgG4 monoclonal antibody was employed (Clone No.: MRQ-44; Manufacturer: Cell Marque, Rocklin, CA, USA; Catalog No.: 276M-96; Dilution ratio: 1:300). The staining procedure followed the EnVision two-step method, with diaminobenzidine (DAB) as the chromogen and hematoxylin as the counterstain. Counting Criteria for IgG+ and IgG4+ Plasma Cells: Three positive cell-rich regions (hot spots) were selected under a high-power field (HPF, ×400) to count IgG+ and IgG4+ plasma cells. The assessment was conducted by two investigators in a double-blind manner, and the average value was used to calculate the IgG4+/IgG+ ratio.

### 2.5. Laboratory Findings

Serum IgG and IgG4 testing was not timely performed due to an initial diagnosis of meningioma. After the second surgery of subtotal resection, an elevated serum IgG4 level (1521 μg/mL;36 μg/mL < normal range < 1350 μg/mL), but normal total IgG level (17,200 μg/mL; 860 μg/mL < normal range < 1740 μg/mL) were detected in the patient.

### 2.6. Treatment and Follow-Up

The patient was postoperatively medicated with prednisone acetate (60 mg/d), calcium supplements and proton pump inhibitors to prevent complications. The follow-up imaging at 3 months postoperatively showed a significant reduction in diffuse meningeal thickening and enhancement, suggesting a sensitive response of IgG4-RHP to glucocorticoid therapy (Figure 2I–L).

## 3. Literature Review on 34 Cases of IgG4-RHP

We searched PubMed and Google Scholar for literature using the keywords “IgG4”, “hypertrophic pachymeningitis”, and “intracranial lesions”. Cases without pathological confirmation and those involving only intraspinal lesions were excluded (Please refer to the Supplementary Materials: File S1: Inclusion and Exclusion Criteria Analysis), and a total of 34 patient case reports were ultimately selected (as shown in Table 1).

A multidimensional characterization analysis was performed on these 34 cases. The patient cohort predominantly consisted of individuals ranging from young to elderly (mean age: 48.6 years, range: 15–86 years), including 25 males (73.5%) and 9 females (26.5%). The main clinical manifestations were as follows: headache in 20 cases (58.8%), seizures in 7 cases (20.6%), visual impairment in 5 cases (14.7%), hearing loss in 5 cases (14.7%), limb sensory disturbance in 4 cases (11.8%), facial sensory disturbance in 3 cases (8.8%), nausea in 3 cases (8.8%), ataxia in 2 cases (5.9%), dysarthria in 2 cases (5.9%), dizziness in 2 cases (5.9%), limb weakness in 1 case 2.9%), scalp mass in 1 case (2.9%), syncope in 1 case 2.9%) and facial mass in 1 case (2.9%). Two patients presented with chronic subdural hematoma on the affected side, and one patient developed superior sagittal sinus venous thrombosis at the central site of the lesion. Among all enrolled cases, only 13 were correctly diagnosed at initial presentation, 8 case reports did not mention the initial diagnosis, and 13 were misdiagnosed initially (misdiagnosis rate: 38.2%). The main misdiagnoses included meningioma, metastatic tumor, tuberculous meningoencephalitis, and meningitis. These findings indicate that IgG4-RHP is associated with a high misdiagnosis rate. Among all enrolled cases, 11 underwent subtotal resection, 19 were subjected to meningeal biopsy, and the surgical approach was not mentioned in 4 cases (as shown in Table 2).

All 34 enrolled cases were confirmed by pathological examination (including cases verified by re-examination), among which 6 patients (17.6%) were misdiagnosed in the initial pathological assessment. Dense lymphoplasmacytic infiltration and storiform fibrosis were identified in all cases (34/34). Obliterative phlebitis was detected in 8 cases, not detected in 9 cases, and not mentioned in 17 case reports. Among the cases, 27 (79.4%) met the criterion of >10 IgG4+ plasma cells per high-power field (HPF), while this indicator was not reported in 7 cases. Twenty-four cases (70.6%) satisfied the standard of an IgG4+/IgG+ plasma cell ratio exceeding 40%, 5 cases did not meet this standard, and 5 cases lacked relevant information (as shown in Table 2).

Among the 34 cases included in this study, the intracranial distribution of lesion sites exhibited distinct anatomical clustering characteristics, with some cases involving multiple sites. In terms of lesion location classification, supratentorial lesions were the most common, accounting for 50.0% (17 cases), mainly including unilateral/bilateral supratentorial regions and lesions involving both the supratentorium and skull base. The skull base/cavernous sinus region was the second most frequent site, involving 14 cases (41.2%), covering the clival dura mater, middle/posterior skull base, cavernous sinus, and anterior skull base. Posterior fossa lesions were observed in 8 cases (23.5%), primarily including lesions of the posterior fossa dura mater and those involving both the posterior fossa and tentorium cerebelli. Skull invasion was relatively rare, occurring in only 5 cases (14.7%), all presenting as supratentorial lesions combined with skull invasion. Mixed supratentorial and infratentorial lesions were the rarest, with only 1 case (2.9%), involving simultaneous bilateral supratentorial and infratentorial dural involvement (as shown in Table 3). One case invaded the retina, and one case invaded the maxillary sinus (as shown in Table 2).

Regarding serum IgG4 levels among the 34 enrolled cases, 11 cases had normal levels (<1350 μg/mL), 11 cases had elevated levels (>1350 μg/mL), and 12 case reports indicated that serum IgG4 levels were not measured.

In terms of treatment, among the 34 enrolled cases, 19 underwent meningeal biopsy, 11 underwent subtotal tumor resection, and the surgical approach was not specified in 4 case reports. Among the 19 patients who underwent meningeal biopsy: 13 achieved favorable outcomes, 5 had no mentioned outcome data, and 1 showed poor response. Among the 11 patients who underwent subtotal tumor resection: 7 obtained favorable outcomes, and 4 had no mentioned outcome data (as shown in Table 1 and Table 2). Postoperatively, 25 cases received pharmacotherapy with the following regimens: 11 cases treated with glucocorticoids alone, 9 cases with glucocorticoids combined with rituximab, 2 cases with glucocorticoids combined with azathioprine, 2 case with glucocorticoids combined with methotrexate, and 1 case with glucocorticoids combined with paclitaxel liposome. Three cases did not receive pharmacotherapy due to poor physical status, and 6 case reports did not mention relevant medication details. Among the 25 patients who received pharmacotherapy, only 1 showed no response to treatment, while the remaining patients achieved significant improvements in both clinical symptoms and imaging findings (as shown in Table 1).

## 4. Discussion

### 4.1. Epidemiology and Clinical Symptoms

IgG4-RHP is an extremely rare autoimmune disorder of the central nervous system (CNS), first reported in 2009 [26], with an incidence of less than 1 per 100,000 population, based on a nationwide survey of hypertrophic pachymeningitis in Japan [27,28]. The incidence is higher in males than in females, with a male-to-female ratio of approximately 2:1, and the mean age at onset is around 52 years. IgG4-RHP is rarely associated with systemic diseases, and more than half of the cases present as isolated lesions [29].

A retrospective study of 33 cases by Lucy X. Lu et al. demonstrated that 22 cases (67%) had headache, 11 cases (33%) were complicated with cranial nerve palsy, 7 cases (21%) had visual abnormalities (diplopia or decreased visual acuity), 5 cases (15%) had limb weakness, 4 cases (12%) were accompanied by limb numbness, 3 cases (9%) had sensorineural hearing loss, and 2 cases (6%) experienced seizures [30]. These results are highly consistent with the statistical findings of the present study. Headache may be caused by dural inflammation or increased intracranial pressure. Cranial nerve palsy and other neurological deficit symptoms are mostly attributed to the compression of brain tissue and cranial nerves by the thickened dura mater, or secondary injuries and complications resulting from vascular compression [14].

### 4.2. Imaging Characteristics

The imaging manifestations of IgG4-RHP are crucial for initial diagnosis. On computed tomography (CT) and magnetic resonance (MR) imaging, it can present as band-like or nodular dural thickening, or mass-like protrusions [30], which are easily confused with Rosai-Dorfman disease, vasculitis, tuberculous meningitis, atypical meningioma, central nervous system leukemia, and other conditions [1]. A positron emission tomography (PET)-CT scan is useful for excluding the involvement of other tissues or organs. Extracranial manifestations can be detected in at least 40% of IgG4-RD patients. Therefore, a thorough systematic examination is necessary to rule out comorbidities such as asymptomatic cirrhosis, pancreatitis, nephritis, aneurysms, and arteritis. Overall, regular imaging during follow-up is preferred for the continuous monitoring of IgG4-RHP [31].

Matias et al. [1] summarized the MRI features of 32 patients with IgG4-RHP and found that “diffuse dural thickening + homogeneous enhancement” is the most typical manifestation, with a specificity of 83% but a sensitivity of only 67%, suggesting that imaging alone is insufficient for a definitive diagnosis. Skull involvement is a rare manifestation of IgG4-RHP. Most reported cases in the literature are associated with skull hyperostosis at the site of lesion involvement [8], while the case reported in this study presents with skull destruction and defects caused by lesion invasion, with proliferative dural tissue extending subcutaneously through the bone defect area. Patients are often misdiagnosed with malignant tumors due to skull erosion or defects observed on CT scans. In addition, cystic lesions [32], tumor-like masses [14], and other special manifestations have been reported, further increasing the difficulty of clinical diagnosis. The present case is characterized by “a history of chronic subdural hematoma + skull destruction + subcutaneous soft tissue mass”, a combination that has not been documented in previous studies. This unique clinical presentation enriches the phenotypic spectrum of IgG4-RHP.

### 4.3. Diagnostic Methods

Meningeal biopsy is the gold standard for diagnosing IgG4-RHP after excluding infectious, neoplastic, and other inflammatory pathologies [16]. Since IgG4-RHP is a rare subtype of IgG4-RD, the pathological diagnostic criteria for IgG4-RHP are identical to those for IgG4-RD.The classic three key histopathological features required for the diagnosis of IgG4-RD, known as the IgG4-RD triad, consist of dense lymphoplasmacytic infiltration, storiform fibrosis (radially arranged fibroblasts/myofibroblasts), and obliterative phlebitis (inflammatory venous occlusion). The diagnosis of IgG4-RD can be confirmed if two or more key histopathological features are present, accompanied by a marked increase in the number of IgG4-positive plasma cells (IgG4+/IgG+ ratio > 40% or >10 IgG4-positive plasma cells per high-power field [HPF, ×400]), especially for meningeal lesions [30]. The case reported herein fits this scenario. The pathological findings fulfilled the criteria of dense lymphoplasmacytic infiltration and storiform fibrosis. Although the IgG4+/IgG+ ratio was approximately 30% (<40%), the number of IgG4-positive plasma cells was elevated (≈40 IgG4+ plasma cells per high-power field [HPF, ×400]). Typical fibrosis or phlebitis may be absent in lymph nodes, lungs, and salivary glands [1]. It should be noted that small biopsy specimens may hinder the detection of obliterative phlebitis or an adequate number of IgG4-positive plasma cells, so a comprehensive assessment combining multi-site biopsy and clinical findings is required.

It is crucial to emphasize that elevated serum IgG4 concentration is not a necessary condition for the diagnosis of isolated IgG4-RHP. Unlike systemic IgG4-RD, due to the relatively low blood circulation in the dural tissue, patients with IgG4-RHP involving only the dura mater may have normal peripheral serum IgG4 concentrations, making them highly susceptible to misdiagnosis as meningitis or meningioma [22]. Therefore, elevated IgG4 levels in cerebrospinal fluid (CSF) are more specific for diagnosis. However, based on the cases analyzed in this study, due to limited clinical awareness of this disease and the invasive nature of lumbar puncture, there are few case reports on CSF IgG4 testing in clinical practice [33]. In addition, CSF examination often shows leukocytosis (WBC > 5/mm^3^) and elevated protein levels (Protein > 40 mg/dL), but these indicators lack specificity [34].

### 4.4. Differential Diagnosis

#### 4.4.1. Meningioma

Meningioma is the most common benign tumor of the central nervous system, accounting for approximately 15% of all intracranial tumors [35]. Its imaging features are similar to those of IgG4-RHP, with both presenting as dural-based masses accompanied by significant enhancement. However, meningiomas are usually well-circumscribed, localized round or oval masses, and calcification can be observed in 20–30% of cases. Pathologically, meningiomas are composed of circumferentially arranged meningothelial cells, without IgG4+ plasma cell infiltration or storiform fibrosis [36]. In the present case, CT showed a slightly hyperdense mass, and MRI revealed dural enhancement. The initial biopsy misdiagnosed it as atypical meningioma. Final pathological examination did not identify meningothelial cell proliferation or psammoma bodies, but showed extensive IgG4+ plasma cell infiltration, which is the key basis for differentiating this disease from meningioma.

#### 4.4.2. Metastatic Tumor

Intracranial dural metastatic tumors are lesions formed by the hematogenous spread, direct extension, or CSF dissemination of malignant tumors to the dura mater. Their magnetic resonance (MRI) imaging features are often focal nodular or massive masses with obvious heterogeneous enhancement and ill-defined boundaries. Lesions are mostly located in the cerebral convexity and parasagittal region, and are often accompanied by erosion or destruction of adjacent skull bones. Clinically, most patients have a history of malignant tumors, and symptoms (such as headache, vomiting, seizures, or focal neurological deficits) progress rapidly. CSF cytological examination may detect tumor cells, but a definitive diagnosis relies on pathological biopsy, which shows atypical tumor cells under microscopy. This disease is insensitive to glucocorticoid therapy or only has a transient response, and treatment requires comprehensive therapies such as radiotherapy and chemotherapy targeting the primary tumor [37].

#### 4.4.3. Tuberculous Meningitis

Tuberculous pachymeningitis is an infectious disease, usually with acute or subacute onset, accompanied by toxic symptoms such as fever and night sweats. Most patients have a history of pulmonary tuberculosis or tuberculosis exposure. The key differentiating point between the two lies in CSF examination: patients with tuberculous pachymeningitis show typical “three highs and one low” changes in CSF (increased pressure, elevated protein, increased cell count, decreased glucose, and decreased chloride). Acid-fast bacilli can be detected in smears or cultures, and PCR testing for Mycobacterium tuberculosis is positive [38]. In contrast, IgG4-RHP is an autoimmune disease with a chronic course, mainly presenting with intractable headache, and often complicated with systemic multi-organ involvement. Significantly elevated serum IgG4 levels are one of the core features of IgG4-RHP, and the disease responds well to glucocorticoid therapy. A definitive diagnosis ultimately depends on pathological biopsy.

### 4.5. Treatment and Prognosis

For cases with intracranial mass effect caused by dural thickening, subtotal tumor resection is often performed to relieve pressure and obtain specimens for pathological examination; for cases without intracranial mass effect, minimally invasive puncture and meningeal biopsy can be performed to confirm the diagnosis. However, the local cerebral tissue compression symptoms in patients who underwent subtotal tumor resection were all significantly relieved after the surgery. After pathological confirmation, most cases are treated with glucocorticoid monotherapy or combined with immunosuppressive therapy, and all achieve favorable prognoses. GCs are the mainstay of treatment for inducing and maintaining remission in patients with IgG4-RD. In the case reported in this study, the patient was administered oral prednisone acetate 60 mg once daily after meals. Concurrently, oral calcium supplements and proton pump inhibitors were prescribed to mitigate the adverse effects of glucocorticoids. Literature reports indicate that lesions in some cases can be completely resolved after pharmacotherapy, but even with glucocorticoid maintenance therapy, the recurrence rate remains relatively high [39]. Data from a study by Kenichi Takano et al. [40] showed that after glucocorticoid treatment and maintenance, the clinical remission rate of patients was 73.8%, the annual recurrence rate was 11.5%, and 50% of cases relapsed within 7 years after initial treatment. If recurrence occurs, it is necessary to increase the dose of glucocorticoids or switch to other immunosuppressive agents (such as rituximab) [38]. Rituximab is an alternative for severe, recurrent, or glucocorticoid-dependent IgG4-RD [28]. Emerging evidence supports the high efficacy of intrathecal rituximab in treating refractory IgG4-RD, with immunological and imaging improvements observed in patients with a poor response to intravenous administration [41].

## 5. Limitations

This study has several limitations that should be acknowledged. First, as a single case report combined with a literature review, the generalizability of conclusions regarding treatment strategies and prognosis is inherently limited. Large-scale prospective studies are therefore warranted to validate the observed findings. Second, the 3-month follow-up duration is relatively short given the known high relapse risk of IgG4-related diseases (with an annual recurrence rate of 11.5% as previously reported). Longer-term clinical and imaging follow-up (≥1 year) is essential to evaluate the sustained efficacy of glucocorticoid therapy and closely monitor for potential recurrence. Third, preoperative serum IgG4 measurement was not performed, which restricts the assessment of its value in early diagnostic decision-making and may have contributed to the initial misdiagnosis. Fourth, cerebrospinal fluid (CSF) IgG4 analysis—an indicator with higher specificity for isolated intracranial IgG4-RHP—was not conducted in this case, reflecting the gap between ideal diagnostic standards and real-world clinical practice. Finally, the literature review presented herein is descriptive rather than quantitative (e.g., meta-analysis). Future studies could employ systematic review and meta-analysis methodologies to further clarify the correlations between pathological thresholds, treatment regimens, and clinical outcomes.

## 6. Conclusions

Histopathological examination remains the gold standard for the definitive diagnosis of IgG4-RHP, as imaging findings and serum IgG4 levels may be nonspecific or overlapping with other intracranial lesions. Surgical intervention, whether subtotal resection or meningeal biopsy, not only provides critical tissue samples for pathological confirmation but also effectively alleviates the mass-occupying effect caused by dural thickening, relieving neurological compression symptoms. Glucocorticoids are the first-line therapeutic option for IgG4-RHP, demonstrating significant efficacy in reducing lesions and improving clinical outcomes, as evidenced by the notable regression of residual lesions in the reported case and most literature-reviewed cases. Given the relatively high recurrence rate of IgG4-RHP, long-term clinical and imaging follow-up is indispensable to timely detect recurrence and adjust treatment strategies. For refractory cases unresponsive to glucocorticoids or those with recurrence, rituximab serves as a reliable alternative therapeutic agent. Additionally, enhancing clinical awareness of atypical manifestations of IgG4-RHP (such as skull destruction and subcutaneous mass formation) and emphasizing the combination of histopathological features, serum IgG4 detection, and clinical context can effectively reduce misdiagnosis rates and optimize patient management.

## Figures and Tables

**Table 1:** Clinical, Pathological, Therapeutic and Outcome Characteristics of 34 Cases.

NO.	Author	Year	Sex/Age	Clinical Manifestations	Core Pathological Features ^1^	Tissue IgG4+ Stain (>10/HPF) ^2^	IgG4+/IgG+ Ratio (>40%) ^3^	Serum IgG4 ^4^ (μg/mL)	Treatment ^5^	Outcome
1	Wallace ZS [4]	2013	F/50	Epilepsy	LPI(+), SF(+), OP(−)	45	90%	No exam	GCs + RTX	Positive
2	Wallace ZS	2013	F/52	Headache, dysarthria, ataxia	LPI(+), SF(+), OP(+)	161	51%	No exam	GCs + RTX	Positive
3	Wallace ZS	2013	M/39	Headache, limb sensory impairment	LPI(+), SF(+), OP(−)	98	70%	No exam	No meds	N/A
4	Lin CK [5]	2013	M/52	Epilepsy	LPI(+), SF(+), OP(−)	30–35	50%	Normal	No meds	N/A
5	Utsuki S [6]	2013	M/37	Headache	LPI(+), SF(+), OP(−)	53	49%	No exam	GCs	Positive
6	Utsuki S	2013	M/43	Headache, diplopia	LPI(+), SF(+), OP(−)	38	43%	No exam	GCs	Positive
7	Utsuki S	2013	M/65	Headache, visual impairment	LPI(+), SF(+), OP(−)	77	63%	No exam	GCs	Positive
8	Utsuki S	2013	M/82	Headache, facial sensory impairment	LPI(+), SF(+), OP(−)	55	67%	No exam	GCs	Positive
9	Rodríguez-Castro E [7]	2015	F/54	Headache, hearing impairment	LPI(+), SF(+), OP(−)	Yes	16%	Normal (917)	GCs	Positive
10	Nambirajan A [8]	2019	M/16	Epilepsy	LPI(+), SF(+), OP(−)	30–35	80%	Normal (1290)	No meds	N/A
11	Levraut M [9]	2019	M/66	Headache, visual impairment	LPI(+), SF(+), OP(+)	Yes	>40%	Normal (401)	GCs + RTX	Positive
12	Ota K [10]	2020	M/56	Headache, diplopia	LPI(+), SF(+), OP(−)	60	50%	No exam	GCs + PL	Positive
13	Zhao Y [2]	2020	M/38	Headache, hearing impairment	LPI(+), SF(+), OP(+)	Yes	>40%	High (1650)	No meds	N/A
14	Zhao Y	2020	M/46	Headache, visual impairment	LPI(+), SF(+), OP(+)	Yes	>40%	High (1430)	No meds	N/A
15	Zhao Y	2020	M/51	Headache, limb sensory impairment	LPI(+), SF(+), OP(+)	Yes	>40%	High (1530)	No meds	N/A
16	Zhao Y	2020	M/41	Headache	LPI(+), SF(+), OP(+)	Yes	>40%	High (1400)	No meds	N/A
17	Suisa H [11]	2021	F/63	Headache, nausea, hearing impairment	LPI(+), SF(+), OP(−)	Yes	40%	High (3480)	GCs + RTX	Positive
18	Yamamuro S [12]	2021	M/51	Diplopia	LPI(+), SF(+), OP(−)	Not mentioned	15.20%	Normal (67)	GCs	Positive
19	Woo PYM [13]	2021	M/86	Headache, visual impairment	LPI(+), SF(+), OP(+)	20	30%	High (2020)	GCs	Positive
20	Esmaeilzadeh M [14]	2022	F/67	Facial sensory impairment	LPI(+), SF(+), OP(−)	Not mentioned	Yes	High (4710)	GCs + RTX	Positive
21	Esmaeilzadeh M	2022	M/30	Epilepsy	LPI(+), SF(+), OP(−)	Not mentioned	Yes	Normal (770)	GCs	Positive
22	Esmaeilzadeh M	2022	F/16	Ataxia, nausea	LPI(+), SF(+), OP(−)	100	90%	Normal (270)	GCs + MTX	Positive
23	Esmaeilzadeh M	2022	M/15	Epilepsy	LPI(+), SF(+), OP(+)	Not mentioned	Yes	Normal (1110)	GCs + RTX	Positive
24	Yu Y [15]	2022	M/40	Headache	LPI(+), SF(+), OP(−)	200	Not mentioned	High (1900)	GCs	Positive
25	Sergio P [16]	2023	F/25	Headache, hearing impairment, diplopia	LPI(+), SF(+), OP(−)	50–60	No	No exam	GCs + RTX	Positive
26	Lichtblau N [17]	2023	M/68	Limb sensory impairment, dysarthria	LPI(+), SF(+), OP(−)	Yes	Not mentioned	High (2253)	GCs + RTX	Positive
27	Gautier F [18]	2023	M/47	Dizziness, hearing impairment	LPI(+), SF(+), OP(−)	Yes	>60%	High (1160)	GCs + RTX	Positive
28	Suezumi K [19]	2024	M/50	Headache, limb weakness	LPI(+), SF(+), OP(−)	Not mentioned	15–20%	No exam	GCs + AZA	Positive
29	Yeo J [20]	2024	M/63	Scalp mass	LPI(+), SF(+), OP(−)	68	22.70%	Normal (96)	GCs + AZA	Positive
30	Zhang Y [21]	2024	M/56	Dizziness, nausea	LPI(+), SF(+), OP(−)	60	60%	No exam	No meds	N/A
31	Chou ML [22]	2024	F/65	Epilepsy, limb sensory impairment	LPI(+), SF(+), OP(−)	20	Not mentioned	Normal (228)	No meds	N/A
32	Silva GED [23]	2024	M/32	Headache, facial mass	LPI(+), SF(+), OP(−)	Not mentioned	Not mentioned	No exam	GCs	Not Positive
33	Lu Y [24]	2025	F/37	Headache, syncope	LPI(+), SF(+), OP(−)	Yes	Not mentioned	Normal (240)	GCs	Positive
34	Khanna S [25]	2025	M/29	Epilepsy	LPI(+), SF(+), OP(−)	Not mentioned	>20%	High (3250)	GCs + MTX	Positive

^1^ Core Pathological Features: LPI = Lymphoplasmacytic infiltration; SF = Storiform fibrosis; OP = Obliterative phlebitis; (+) = Present, (−) = Absent. ^2^ Some cases only provide positive results without specifying specific numerical values. ^3^ Some cases only provide positive results without specifying specific numerical values. ^4^ Serum IgG4: Normal ≤ 1350 μg/mL, High ≥ 1350 μg/mL, No exam = Not tested; Exact values in parentheses. ^5^ Treatment: GCs = Glucocorticoids; RTX = Rituximab; MTX = Methotrexate; AZA = Azathioprine; PL = Paclitaxel Liposome; No meds = No medication; Not mentioned = Not reported.

**Table 2:** Supplementary Clinical and Surgical Details.

Indicator Category	Case Grouping	Details
Misdiagnosis	Correct (*n* = 13)	Cases 11–16, 20–23, 26–27, 32
	Meningioma and Other specific misdiagnoses (*n* = 13)	Cases 10, 17–18, 30–31 (Meningioma); Case 1 (Tolosa-Hunt syndrome); Case 2 (Etiology unknown); Case 3 (pachymeningitis); Case 4 (Pachymeningitis; Metastatic tumor); Case 19 (Giant cell arteritis); Case 24 (Tuberculous meningitis; Meningioma); Case 25 (Inflammatory myofibroblastic tumor); Case 28 (Brain tumor)
	Not mentioned (*n* = 8)	Cases 5–9, 29, 33, 34
Surgical Method	Subtotal resection (*n* = 11)	Cases 1, 4, 10, 17–18, 21, 23, 29–31, 34
	Meningeal biopsy *(n* = 19)	Cases 2–3, 9, 11–16, 19–20, 22, 24–28, 32, 33
	Not mentioned (*n* = 4)	Cases 5–8
Initial Pathology Correct	Correct (*n* = 25)	Cases 10–14, 16–24, 26–30, 32–34
	Incorrect (*n* = 6)	Cases 1–4, 25, 31
	Not mentioned (*n* = 3)	Cases 5–9
Extracranial extension	Number (*n* = 2)	Cases 19 (Retina); Cases 32 (maxillary sinus)
Pathological changes accompanying the nervous system	Number (*n* = 3)	Cases 12, 26 (Chronic subdural hematoma); Cases 33 (Superior sagittal sinus thrombosis)

Case numbering aligns with Table 1; *n* = number of cases in each subgroup. “Initial Pathology Correct”: “Not mentioned” = No data reported. Lesion site subgroups may overlap (e.g., some cases involve both supratentorial and skull base regions). All details are consistent with original clinical and surgical data.

**Table 3:** Statistics of intracranial disease locations.

Lesion Site Category	Included Specific Sites	Case Number (*n*)	Percentage (%)	Corresponding Case No.
Supratentorial	Left/right/bilateral supratentorial; supratentorial + skull base	17	50.0	1, 3–4, 7–8, 10, 11, 16, 19, 26, 28–31, 33, 34
Posterior fossa	Posterior fossa dura mater; posterior fossa + tentorium cerebelli	8	23.5	2, 9, 14, 22, 25, 27
Skull base/cavernous sinus	Clival dura mater; middle/posterior skull base; cavernous sinus; anterior skull base	14	41.2	5–6, 9, 11, 13, 15, 17–18, 20–21, 23–25
Skull invasion	Supratentorial + skull invasion	5	14.7	4, 28–29, 34
Mixed sites (supra+infra)	Bilateral supratentorial and infratentorial	1	2.9	12

Data are presented as number of cases (*n*) and percentage (%). Some cases involve multiple lesion sites, so the total number of involved cases exceeds 34. Case numbering aligns with previous clinical characteristic tables.

## Data Availability

The data supporting the findings of this study are available from the corresponding author upon reasonable request, as ethical restrictions prevent public deposition of individual patient data.

## Supplementary Materials

The following supporting information can be downloaded at: https://www.mdpi.com/article/10.3390/diagnostics16050682/s1, File S1: Inclusion and Exclusion Criteria Analysis.

## Abbreviations

The following abbreviations are used in this manuscript:

IgG4	immunoglobulin G subclass 4
IgG	immunoglobulin G
CNS	central nervous system
CT	computed tomography
MRI	magnetic resonance imaging
HPF	high-power field
IgG4-RD	IgG4-related disease
IgG4-RHP	IgG4-related hypertrophic pachymeningitis
PET	positron emission tomography
HP	hypertrophic pachymeningitis
CSF	cerebrospinal fluid
GCs	Glucocorticoid
RTX	Rituximab
MTX	Methotrexate
AZA	Azathioprine
PL	Paclitaxel Liposome
LPI	Lymphoplasmacytic infiltration
SF	Storiform fibrosis
OP	Obliterative phlebitis
HU	Hounsfield unit
