# Supplementary material for: IgG4-Related Disease Manifested as Hypertrophic Pachymeningitis: A Case Report and Literature Review
Source: Diagnostics (Basel). 2026 Feb 26;16(5):682. doi: 10.3390/diagnostics16050682 (PMC12984918; doi:10.3390/diagnostics16050682)
Supplement: Supplementary file 1 [file diagnostics-16-00682-s001.zip › diagnostics-4122635-supplementary.pdf]

Summary of Literature Published in 2025–2026 & Before 2025: Inclusion and Exclusion Criteria Analysis

1. Takano K. Head and neck manifestations of IgG4-related disease: current understanding[J]. Japanese Journal of Radiology, 2025, https://doi.org/10.1007/s11604-025-01934-y.

Main content: Focuses on the head and neck manifestations of IgG4-related disease, with an emphasis on IgG4-related dacryoadenitis and sialadenitis, covering key aspects such as clinical features, diagnostic criteria, imaging findings, and differential diagnosis.

Inclusion/Exclusion Reason: Excluded. This literature primarily focuses on the head and neck manifestations of IgG4-related disease, while our manuscript centers on isolated central nervous system (CNS) IgG4-related disease (without systemic involvement) or further local manifestations caused by extracranial invasion of intracranial dura mater. Such cases are more prone to misdiagnosis compared to those of IgG4-related disease complicated with intracranial hypertrophic pachymeningitis.

2. Yang L, Kaninia S, Urankar K, et al. Cerebral venous sinus thrombosis complicating IgG4-related hypertrophic pachymeningitis[J]. Practical Neurology, 2025, pn-2025-004845, https://doi.org/10.1136/pn-2025-004845

Main content: Reports a case of IgG4-related hypertrophic pachymeningitis complicated with cerebral venous sinus thrombosis, detailing symptoms, examination results, and symptom relief after treatment with hormones and other therapies.

Inclusion/Exclusion Reason: Included. This is an interesting case that we have incorporated into our review. The literature enriches the clinical manifestations and special presentation forms of the disease.

3. Kim B, Lee KM. Intracranial Immunoglobulin G4-Related Disease Presented as Hypophysitis and Pachymeningitis: A Case Report[J]. J Korean Soc Radiol, 2025, 86(3):425-431, https://doi.org/10.3348/jksr.2024.0096.

Main content: Reports a case of intracranial IgG4-related disease presenting as hypophysitis and pachymeningitis, describing its clinical symptoms, serum and imaging examinations, and improvement after hormone therapy.

Inclusion/Exclusion Reason: Excluded. This patient lacks histopathological diagnostic evidence.

4. Ansari S, Mu BH, Findley J, et al. Case 342: IgG4-related Hypertrophic Pachymeningitis[J]. Radiology, 2025, 317(1):e243264, https://doi.org/10.1148/radiol.243264.

Main content: Presents a case of spinal IgG4-related hypertrophic pachymeningitis, describing the patient's symptoms, imaging and pathological features, as well as favorable outcomes after treatment with glucocorticoids combined with rituximab.

Inclusion/Exclusion Reason: Excluded. Initially, we also collected many cases of isolated IgG4-related spinal hypertrophic pachymeningitis. However, considering that the initial symptoms of these patients are significantly different from those with isolated CNS IgG4-related disease (without systemic involvement), we plan to separately review patients with isolated IgG4-related spinal hypertrophic pachymeningitis. This may better help clinicians avoid confusion and misdiagnosis.

5. Wang J, Li W, Zeng L, et al. Hypertrophic Pachymeningitis Mimicking Hemorrhage[J]. Neurol India, 2025, 73:1118-1119, https://doi.org/10.4103/neurol-india.Neurol-India-D-25-00175.

Main content: Reports a case of hypertrophic pachymeningitis mimicking hemorrhage, with the patient presenting with symptoms such as headache. The symptoms stabilized after imaging examinations and high-dose methylprednisolone treatment.

Inclusion/Exclusion Reason: Excluded. This patient does not have IgG4-related hypertrophic pachymeningitis。

6. Khanna S, Yadav S, Balakrishnan C. Intracranial IgG4-related Disease: Insights from Two Cases[J]. Journal of The Association of Physicians of India, 2025, 73(11):54-56, https://doi.org/10.59556/japi.73.1244.

Main content: Reports two cases of intracranial IgG4-related disease, presenting as isolated meningitis and multisystem hypophysitis respectively, suggesting that diagnosis requires flexibility, and glucocorticoids combined with methotrexate are effective.

Inclusion/Exclusion Reason: Included. This literature reports two cases. Case 1: Clinical manifestations of isolated CNS IgG4-related disease (without systemic involvement). Case 2: Although presenting with CNS symptoms,

PET-CT showed multisystem involvement. Case 2 is not within the statistical scope of our manuscript, and we have included Case 1 in our review.

7. Chodankar NU, Dhupar V, Dhupar A, et al. Immunoglobulin G4 related sclerosing disease mimicking a lytic lesion of the mandible: a case report and review of literature[J]. Oral and Maxillofacial Surgery, 2025, 29:24, https://doi.org/10.1007/s10006-024-01325-z.

Main content: Reports a case of IgG4-related sclerosing disease presenting as a lytic lesion of the mandible, diagnosed by serological and pathological examinations. The lesion completely resolved 2 years after oral hormone treatment.

Inclusion/Exclusion Reason: Excluded. The patient only has IgG4-related sclerosing disease with a lytic lesion of the mandible, which is unrelated to intracranial meningitis.

8. Salmaggi A, Cesana C, Piparo M, et al. A case of IgG4RD presenting as spinal compression[J]. Neurological Sciences, 2025, 46:5533-5536, https://doi.org/10.1007/s10072-025-08334-0.

Main content: Reports a case of a 57-year-old male with IgG4-related disease causing cervicothoracic spinal cord compression. Serum IgG4 was normal, and the diagnosis was confirmed by pathology. Clinical and imaging improvements were achieved after hormone treatment.

Inclusion/Exclusion Reason: Excluded. Initially, we also collected many cases of isolated IgG4-related spinal hypertrophic pachymeningitis. However, considering that the initial symptoms of these patients are significantly different from those with isolated intracranial manifestations, we plan to separately review patients with isolated CNS IgG4-related disease (without systemic involvement). This may better help clinicians avoid confusion and misdiagnosis.

9. Alshoumer AS, Alyamany BM, Alanazi LT. A Rare Presentation of a Spinal Lesion: Immunoglobulin G4-Related Hypertrophic Spinal Pachymeningitis[J]. Cureus, 2025, 17(4):e83120, https://doi.org/10.7759/cureus.83120.

Main content: Reports a case of a 64-year-old male with IgG4-related hypertrophic spinal pachymeningitis, presenting as cervical spinal cord compression. Symptoms improved after surgical resection combined with hormone treatment, and the diagnosis was confirmed by pathology.

Inclusion/Exclusion Reason: Excluded. Initially, we also collected many cases

of isolated IgG4-related spinal hypertrophic pachymeningitis. However, considering that the initial symptoms of these patients are significantly different from those with isolated intracranial manifestations, we plan to separately review patients with isolated CNS IgG4-related disease (without systemic involvement). This may better help clinicians avoid confusion and misdiagnosis.

10. Gavioli F, Capogna A, Terribili R, et al. IgG4-related disease presenting as hypertrophic pachymeningitis[J]. Clinical Rheumatology, 2025, https://doi.org/10.1007/s10067-025-07585-8.

Main content: Reports a case of a 66-year-old female with IgG4-related disease presenting as hypertrophic pachymeningitis, accompanied by a thoracic epidural mass and spinal cord compression. The condition improved after treatment with rituximab combined with hormones.

Inclusion/Exclusion Reason: Excluded. Initially, we also collected many cases of isolated IgG4-related spinal hypertrophic pachymeningitis. However, considering that the initial symptoms of these patients are significantly different from those with isolated intracranial manifestations, we plan to separately review patients with isolated CNS IgG4-related disease (without systemic involvement). This may better help clinicians avoid confusion and misdiagnosis.

11. Guo LJ, Li CJ, Wei H, et al. Clinical analysis of immunoglobulin G4-related disease with neurological involvement[J]. Chin J Intern Med, 2025, 64(10):963-970, https://doi.org/10.3760/cma.j.cn112138-20241108-00742.

Main content: Retrospectively analyzes 9 patients with IgG4-related disease involving the nervous system. Hypertrophic pachymeningitis is the most common, hormone treatment is effective but with a high recurrence rate, and early combination of immunosuppressants can improve prognosis.

Inclusion/Exclusion Reason: Excluded. IgG4-related disease is relatively not rare, and many patients have the same neurological lesions. However, such patients often have many clinical symptoms or abnormal biochemical examinations of IgG4-related disease, and the proportion of misdiagnosed patients is not high. Our manuscript focuses on isolated CNS IgG4-related disease (without systemic involvement), which is highly prone to misdiagnosis due to confusion with meningioma, tuberculous meningoencephalitis, etc. Our manuscript aims to enable clinicians to consider this rare condition when seeing imaging data of patients with isolated CNS IgG4-related disease (without systemic involvement) occurring solely in the intracranial region, thereby reducing the rate of misdiagnosis. Our review also confirms that many

clinicians have misdiagnosed such cases.

12. Ng YZJ, Bowen S, Phillips J, et al. Neurological manifestations of immunoglobulin G4 related disease: a systematic review of 393 cases[J]. J Neurol Neurosurg Psychiatry, 2025, 96:1109-1116, https://doi.org/10.1136/jnnp-2025-336230.

Main content: A systematic review of 393 cases found that hypertrophic pachymeningitis, orbital disease, and hypophysitis are the most common neurological manifestations of IgG4-RD. Glucocorticoids are the first-line treatment, and rituximab is an important second-line option.

Inclusion/Exclusion Reason: Excluded. IgG4-related disease is relatively not rare, and many patients have the same neurological lesions. However, such patients often have many clinical symptoms or abnormal biochemical examinations of IgG4-related disease, and the proportion of misdiagnosed patients is not high. Our manuscript focuses on isolated CNS IgG4-related disease (without systemic involvement), which is highly prone to misdiagnosis due to confusion with meningioma, tuberculous meningoencephalitis, etc. Our manuscript aims to enable clinicians to consider this rare condition when seeing imaging data of patients with isolated CNS IgG4-related disease (without systemic involvement) occurring solely in the intracranial region, thereby reducing the rate of misdiagnosis. Our review also confirms that many clinicians have misdiagnosed such cases.

13. Ang T, Kundua N, Patel S, et al. Non-infectious hypertrophic pachymeningitis associated with orbital inflammatory disease: a pooled analysis[J]. ORBIT, 2025, 44(1):49-58, https://doi.org/10.1080/01676830.2024.2390609.

Main content: Pooled analysis of 37 cases of non-infectious hypertrophic pachymeningitis combined with orbital inflammatory disease. Common manifestations include orbital pain and headache. There are no specific imaging markers, so potential etiologies should be vigilant, and treatment often requires a combination of immunosuppressants.

Inclusion/Exclusion Reason: Excluded.

(1) IgG4-related disease is relatively not rare, and many patients have the same neurological lesions. However, such patients often have many clinical symptoms or abnormal biochemical examinations of IgG4-related disease, and the proportion of misdiagnosed patients is not high. Our manuscript focuses on isolated CNS IgG4-related disease (without systemic involvement), which is highly prone to misdiagnosis due to confusion with meningioma, tuberculous meningoencephalitis, etc. Our manuscript aims to enable clinicians to consider

this rare condition when seeing imaging data of patients with isolated CNS IgG4-related disease (without systemic involvement) occurring solely in the intracranial region, thereby reducing the rate of misdiagnosis. Our review also confirms that many clinicians have misdiagnosed such cases.

(2) Hypertrophic pachymeningitis has many etiologies. Based on the case we encountered, our manuscript focuses on IgG4-related hypertrophic pachymeningitis, aiming to share our experience with clinicians and reduce the rate of misdiagnosis.

14. Brescovit DO, Lucato LT, Castro LHM, et al. “Eiffel-by-night” sign in hypertrophic pachymeningitis: Clinical and radiological correlates[J]. Clinical Neurology and Neurosurgery, 2026, 262:109291, https://doi.org/10.1016/j.clineuro.2025.109291.

Main content: Studies 45 patients with hypertrophic pachymeningitis, 17.7% of whom had the "Eiffel-by-night" sign, which is more common in idiopathic cases and associated with disease recurrence. The persistent presence of this sign indicates a high risk of recurrence.

Inclusion/Exclusion Reason: Excluded. Hypertrophic pachymeningitis has many etiologies. Based on the case we encountered, our manuscript focuses on isolated CNS IgG4-related disease (without systemic involvement), aiming to share our experience with clinicians and reduce the rate of misdiagnosis.

15. Balasubramanian K, Patel P, Fassina GR, et al. Idiopathic hypertrophic pachymeningitis masquerading as CNS neoplasm: case report and literature review[J]. Immunologic Research, 2026, 74:1, https://doi.org/10.1007/s12026-025-09738-y.

Main content: Reports a case of idiopathic hypertrophic pachymeningitis mimicking a CNS neoplasm. Combined with a literature review, diagnostic and therapeutic schemes are proposed, emphasizing the importance of biopsy and long-term hormone treatment.

Inclusion/Exclusion Reason: Excluded. This patient does not have IgG4-related hypertrophic pachymeningitis. Hypertrophic pachymeningitis has many etiologies. Based on the case we encountered, our manuscript focuses on isolated CNS IgG4-related disease (without systemic involvement), aiming to share our experience with clinicians and reduce the rate of misdiagnosis.

In addition, the following literatures are excluded for the same or similar reasons：

16. Rayamajhi S, Shrestha R, Sunuwar N, et al. A rare case report on hypertrophic pachymeningitis: Serum IgG4-related disease[J]. Radiology Case Reports, 2022, 17(8): 4371-4375.
17. Martín-Nares E, Hernández Molina G, Baenas DF, et al. Performance of the 2019 ACR/EULAR Classification Criteria for IgG4-Related Disease in a Latin American Cohort[J]. Journal of Clinical Rheumatology, 2024, 30(2): 52-57.
18. Aragonès JM, Arias-Rivero M, García-Barrionuevo JM, et al. IgG4-and MPO-ANCA-associated hypertrophic pachymeningitis[J]. Revista de neurología, 2015, 61(10): 454-457.
19. Takano K, Yamamoto M, Takahashi H, et al. Recent advances in knowledge regarding the head and neck manifestations of IgG4-related disease[J]. Auris Nasus Larynx, 2016, 43(6): 603-613.
20. Della-Torre E, Galli L, Franciotta D, et al. Diagnostic value of IgG4 Indices in IgG4-Related Hypertrophic Pachymeningitis[J]. Journal of Neuroimmunology, 2014, 266(1-2): 82-86.
21. Lu LX, Della-Torre E, Stone JH, et al. IgG4-Related Hypertrophic Pachymeningitis: Clinical Features, Diagnostic Criteria, and Treatment[J]. JAMA Neurology, 2014, 71(6): 785-793.
22. Tuttolomondo A, Simonetta I, Di Raimondo D, et al. IgG4-related syndrome: Another multiorgan disease of internal medicine interest[J]. Current Pharmaceutical Design, 2016, 22(30): 4669-4685.
23. Zhang L, Liu T, Luo J, et al. IgG4-related spinal pachymeningitis[J]. Clinical Rheumatology, 2016, 35(4): 977-983.
24. Lindstrom KM, Cousar JB, Lopes MBS. IgG4-related meningeal disease: clinico-pathological features and proposal for diagnostic criteria[J]. Acta Neuropathologica, 2010, 120(6): 765-776.
25. Della-Torre E, Bozzolo EP, Passerini G, et al. IgG4-related pachymeningitis: evidence of intrathecal IgG4 on cerebrospinal fluid analysis[J]. Annals of Internal Medicine, 2012, 156(5): 401-403.
26. Terrim S, Mahler JV, Marques Filho FV, et al. Clinical Presentation, Investigation Findings, and Outcomes of IgG4-Related Pachymeningitis: A Systematic Review[J]. JAMA Neurology, 2024, doi:10.1001/jamaneurol.2024.3947.
27. Cação G, Calejo M, Alves JE, et al. Clinical features of hypertrophic pachymeningitis in a center survey[J]. Neurological Sciences, 2019, 40(3):543-551.
28. Nomura S, Shimojima Y, Kondo Y, et al. Hypertrophic pachymeningitis in polyarteritis nodosa: a case-based review[J]. Clinical Rheumatology, 2022, 41(2):567-572.
29. Matias TB, Cordeiro RA, Duarte JA, et al. Immune-Mediated Hypertrophic Pachymeningitis and its Mimickers: Magnetic Resonance Imaging Findings[J]. Academic Radiology, 2023, 30(11):2696-2706.
30. Wang Y, Yang Y, Guo X, et al. Clinical and prognostic profiles in immune-mediated hypertrophic meningitis: a retrospective analysis of 92 cases[J]. Clinical Rheumatology, 2025, 44(11):3073-3081.

31. Ikeda J, Shimojima Y, Yamada A, et al. Quantitative evaluation of the thickened dura mater impacting clinical signs in immune-mediated hypertrophic pachymeningitis[J]. Journal of the Neurological Sciences, 2023, 453:120780.
32. Chae TS, Kim DS, Kim GW, et al. Immunoglobulin G4-related spinal pachymeningitis: A case report[J]. World Journal of Clinical Cases, 2024, 12(32):6551-6558.
33. Abe F, Michishita Y, Saito M, et al. Refractory IgG4-related disease complicated with organising pneumonia and hypertrophic pachymeningitis[J]. Modern Rheumatology Case Reports, 2022, 6(2):278-281.
34. Fain O, Mekinian A. Les pachyméningites[J]. La Revue de médecine interne, 2017,
35. Balaban DT, Hutto SK, Panzarini BP, et al. Treatment of IgG4-related disease-associated hypertrophic pachymeningitis with intrathecal rituximab: a case report[J]. Frontiers in Neurology, 2023, 14:1189778.
36. Yonekawa T, Murai H, Utsuki S, et al. A nationwide survey of hypertrophic pachymeningitis in Japan[J]. J Neurol Neurosurg Psychiatry, 2013,
37. De Virgilio A, de Vincentiis M, Inghilleri M, et al. Idiopathic hypertrophic pachymeningitis: an autoimmune IgG4-related disease[J]. Immunology Research, 2016.
